# Effect of Vaccines and Antivirals during the Major 2009 A(H1N1) Pandemic Wave in Norway – And the Influence of Vaccination Timing

**DOI:** 10.1371/journal.pone.0030018

**Published:** 2012-01-10

**Authors:** Birgitte Freiesleben de Blasio, Bjørn G. Iversen, Gianpaolo Scalia Tomba

**Affiliations:** 1 Department of Biostatistics, Institute of Basic Medical Science, University of Oslo, Oslo, Norway; 2 Division of Infectious Disease Control, Norwegian Institute of Public Health, Oslo, Norway; 3 Department of Mathematics, University of Rome “Tor Vergata”, Roma, Italy; 4 Norwegian Computing Center, Oslo, Norway; Mayo Clinic, United States of America

## Abstract

To evaluate the impact of mass vaccination with adjuvanted vaccines (eventually 40% population coverage) and antivirals during the 2009 influenza pandemic in Norway, we fitted an age-structured SEIR model using data on vaccinations and sales of antivirals in 2009/10 in Norway to Norwegian ILI surveillance data from 5 October 2009 to 4 January 2010. We estimate a clinical attack rate of approximately 30% (28.7–29.8%), with highest disease rates among children 0–14 years (43–44%). Vaccination started in week 43 and came too late to have a strong influence on the pandemic in Norway. Our results indicate that the countermeasures prevented approximately 11–12% of potential cases relative to an unmitigated pandemic. Vaccination was found responsible for roughly 3 in 4 of the avoided infections. An estimated 50% reduction in the clinical attack rate would have resulted from vaccination alone, had the campaign started 6 weeks earlier. Had vaccination been prioritized for children first, the intervention should have commenced approximately 5 weeks earlier in order to achieve the same 50% reduction. In comparison, we estimate that a non-adjuvanted vaccination program should have started 8 weeks earlier to lower the clinical attack rate by 50%.

In conclusion, vaccination timing was a critical factor in relation to the spread of the 2009 A(H1N1) influenza. Our results also corroborate the central role of children for the transmission of A(H1N1) pandemic influenza.

## Introduction

The novel A(H1N1) influenza virus, that was identified from an epidemic in Mexico in March 2009, rapidly spread throughout the world and was declared a pandemic (Phase 6) by the WHO on June 11 2009. It became clear at an early stage that the pandemic was relatively mild with considerable immunity against the virus in older people [1]. However, the pandemic was distinct from seasonal influenza as young people and people without any predisposing underlying disease were disproportionately affected in terms of hospitalizations and deaths, and intensive care units were pressured by cases of acute respiratory distress syndrome (ARDS) [2]. Serological and clinical studies indicate that a larger proportion of infections were asymptomatic or mild compared to interpandemic influenza [3], [4].

In Europe, the major pandemic wave hit in October to December, in some countries it arrived following a minor summer wave. The 2009 A (H1N1) pandemic was the first pandemic where antivirals and vaccines were available and it is therefore of great interest to evaluate the effectiveness of these intervention measures. The intervention strategies and national plans for pandemic influenza differed among countries and there was heterogeneity in the severity and timing of the pandemic across the region. Norway holds an extensive list of population based registries including a vaccine registry and a prescription registry based on person numbers (unique person identifiers). Norwegian public health authorities had made an agreement of advance purchase of 9.4 million doses of the adjuvanted vaccine Pandemrix® (Glaxo Smith Kline Biologicals s.a.) in the event of a pandemic. The purchase was later downsized to 6.4 million doses and amounted to 112 million USD (January 2010). The contract secured that Norway would receive the first vaccine produced, and delivery of Pandemrix commenced in October in the midst of the influenza pandemic. The vast majority of vaccinations were carried out between October and December, and data from the vaccine registry show that 1.95 million persons were vaccinated, corresponding to 40% of the population. In addition, some 0.2–0.3 million persons were vaccinated without being registered [5].

Since 2005 , Norway had stockpiled 1.4 million treatment courses of the antiviral Tamiflu® (Roche) and 0.2 million treatment courses of Relenza®(GlaxoSmithKline Biologicals s.a.) due to fear of a bird flu pandemic, in accordance with WHO recommendations. Norway, together with UK, had a liberal policy regarding the use of antivirals, while most countries restricted the prescription of antivirals to doctors only. During the pandemic, Norwegian pharmacists were allowed the temporary right to issue prescriptions of antivirals to ease pressure on health care services.

We estimate the effectiveness of vaccination and antivirals in mitigating the 2009 A(H1N1) influenza in Norway. To this purpose, we developed an age-structured dynamic model that was fitted to surveillance data. The model was used to perform contrafactual simulations to estimate the disease burden of an unmitigated pandemic and also to study the influence of vaccination timing and strategy on the disease burden.

## Methods

### 1. Data

Data from the Norwegian general practitioner (GP) sentinel network was obtained from the Norwegian Institute of Public Health (NIPH). The sentinel network consists of about 200 GPs throughout the country, covering around 15% of the population. During the winter season, from week 40 to week 20, the sentinel GPs report weekly on the proportion of their patient contacts that are given the diagnosis of influenza-like illness (ILI) based on symptoms such as abrupt onset of fever accompanied by respiratory signs and muscle pain. Since 1999 the ILI surveillance is stratified in four age groups: 0–4 years, 5–14 years, 15–64 years and 65+years. In 2009 the surveillance was extended to cover the summer period due to the emerging pandemic.

We obtained age-specific data on the pandemic vaccine coverage from week 43/2009 through week 9/2010 from the Norwegian National Vaccine Registry (SYSVAK). Doctors and nurses were required to register pandemic vaccination by person number and date of vaccination. We used data on purchased antivirals, Tamiflu® and Relenza® from 1 January to 31 December 2009 from the Norwegian Prescription Database (NorPD) containing information on all delivery of medicine in Norway from pharmacies with person identification number and date. Both Tamiflu® and Relenza® are prescription-only preparations in Norway. We stratified data on use of vaccine and antivirals into 3 age groups: 0–14 years, 15–64 years and 65+ years in accordance with the ILI surveillance data.

### 2. Influenza Model

**Figure 1:**
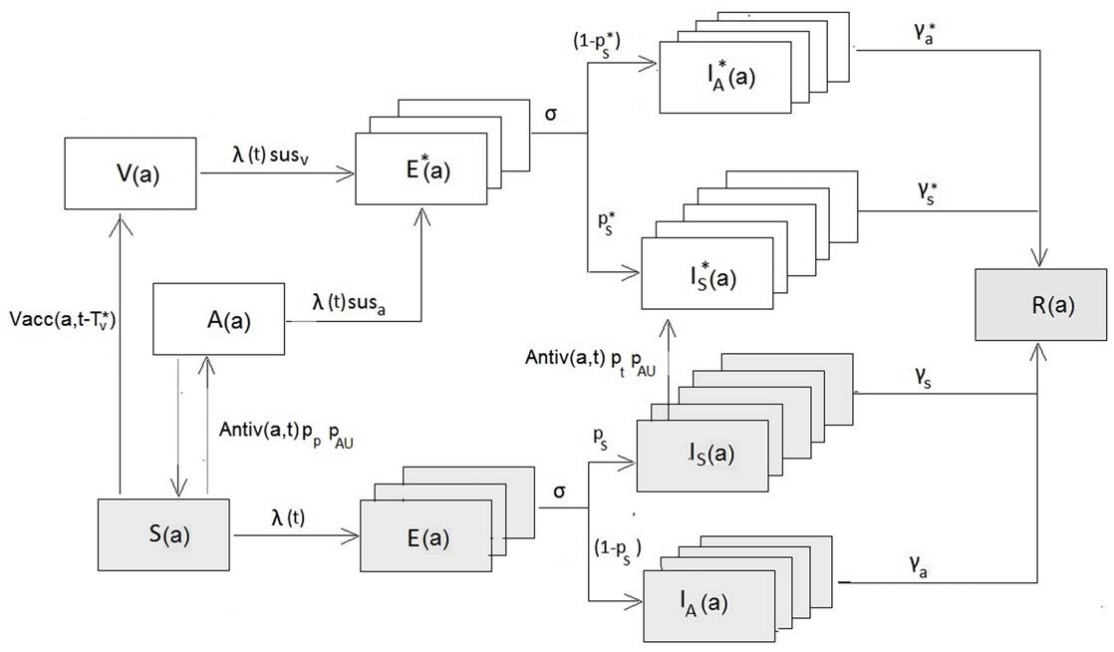
Schematics of the dynamic transmission model for influenza (shaded compartments), with vaccine and antiviral intervention (unshaded).

We used an *SEIR (Susceptible - Exposed (latent) - Infective - Removed)* transmission model with age groups, staged latent and infective periods and additional states for symptoms , vaccination and antiviral treatment status to simulate the dynamics of the 2009 A (H1N1) influenza pandemic (see Figure 1) . In this section we describe the basic structure and parameterization of the model. Details of the model structure and parameterization related to antivirals and vaccination are described in the corresponding sections below. We considered a closed population of size N = 4.868 million, consistent with the Norwegian population in January 2010, divided into sub-populations of children 0–14 years (18,9%), adults 15–64 years (66,4%) and elderly 65+years (14,7%). For the age groups *a* = 1, 2, 3, the Infective class was subdivided into symptomatic infected (*I_S_(a)*) and asymptomatic infected (*I_A_(a)*).

We employed a WAIFW matrix (“Who-acquires-infection-from-whom” matrix) based on the relative contact rates between age groups using empirical contact data from a European study [6]. School-children were considered to have distinct susceptibility (*sus_c_*) and infectivity (*inf_c_*) to influenza to account for potential higher transmissibility in this age group compared to the general population. These parameters, as well as the effective reproductive number 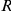, were estimated during the model fitting procedure. The effective reproductive number of the disease was calculated as the largest eigenvalue of the next generation matrix incorporating all assumptions about exposed and infectious stages in the model [7].

To obtain realistic representations of the exposed and infectious periods, we divided these periods into 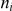 stages (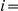 E or I), where the progression from each stage occurs at rate 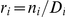, where 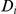 is the average duration of period 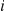. This results in gamma distributed sojourn times with shape parameters 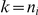 and scale parameters 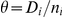. The mean duration of the exposed (incubation) period was set to 1.9 days [8], [9], and modeled in 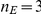 stages. Individuals in the last exposed stage were assumed to be infectious with infectivity 25% compared to full infectivity of symptomatic infections, as viral shedding of influenza increases during the first day following transmission [10]. We further assumed that the proportions 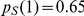 of children and 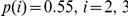 of adults and elderly would develop symptomatic infection [11]. Symptomatic infection was modeled with a mean duration of 5 days with 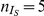 stages. The infectivity in the stages was assumed to be 100%, 100%, 50%, 50% and 25% to give a reasonable infectivity profile in agreement with empirical data showing that viral influenza shedding peaks during the early period after symptoms develop [10]. We set the average duration of the remaining 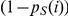 asymptomatic infections to 5 days modeled in 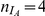 stages. The peak infectivity of asymptomatic infections was assumed to be 50% of the peak level of a symptomatic infection [12], and with 100%, 100%, 50% and 25%, infectivity in the stages, respectively. All infected individuals were assumed to be protected against re-infection during the course of the simulation.

Norwegian sera collected in August 2009 showed low prevalence (3.2%; HI
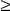
40) of protective antibodies reactive to the 2009 A (H1N1) influenza, which was similar to the pre-pandemic August 2008 levels [13], and we assumed that all children and adults were susceptible to A(H1N1) pandemic influenza at the start of the simulation. Guided by preliminary simulations, we assumed that 60% of the elderly population had pre-existing humoral immunity against the 2009 pandemic influenza. This estimate is somewhat high compared to Norwegian seroprevalence data, but in line with data from Finland showing that 96% of people born between 1909 and 1919, and 77% to 14% of people born between 1920 and 1944 had pre-existing antibodies against the 2009 A (H1N1) influenza [14].

The simulations were performed in Matlab R2010 using the ode45 solver with daily output. On the first day of the simulation a single infected individual in each age group was introduced into all three age groups. The final epidemic size was evaluated by the end of week 3 in 2010 for the fitted models, and by the end of week 12 in scenarios addressing vaccination timing.

### 3. Antivirals

To model the effect of antivirals, we assumed that an overall proportion 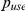 of purchased antivirals were used by people for whom antiviral treatment was intended, and that all individuals in this group followed the recommended treatment schedules. We assumed that a proportion 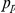 of the antivirals were used for prophylaxis, while the remaining proportion 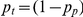 was used for treatment by symptomatic infected individuals. The parameters 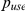 and 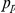 were considered to be constant and age-independent during the course of the pandemic.

A Norwegian population-based internet study among people aged 18–67 years found that 37% (46/123) of people who bought antivirals in the period November 2009 to June 2010 did this with the purpose of private stockpiling [15]. During the major wave, 50–70% of the laboratory tests were positive for influenza [16], and we used this number as a proxy for the proportion of people using antiviral drugs for treatment, who actually experienced an influenza infection. Based on these figures, we made a rough estimate of the proportion of antivirals used effectively 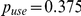 (being approximately equal to (1−0.37)
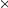
0.6). The use of antivirals for prophylaxis was limited and mainly allocated to high-risk healthcare workers and emergency services personnel at hospitals who had failed to use recommended personal protective equipment [17], and we thus assumed that 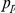 varied between 0.025 and 0.055.

Susceptible individuals 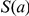 initiating antiviral treatment were moved to states of temporary antiviral protection 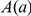; the mean duration of protection was set to 10 days in accordance with prophylaxis treatment guidelines [18]. The antiviral efficacy for susceptibility (ability to prevent infection) was assumed to be 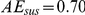, for infectiousness (ability to reduce transmission) 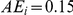, and for pathogenicity (ability to reduce symptoms conditional on infection) 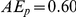, based on data from household studies [19], [20]. The exposed and infective compartments 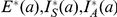 for people undergoing chemoprophylaxis were modeled with similar infectivity profile and number of stages as those used for untreated groups (Table 1), except that the mean duration of the infectious period for people undergoing treatment was reduced by 1 day, following suggestions by Longini et al. [12].

**Table 1:** Model parameters with baseline values, range and distributional assumptions for varied parameters†, and estimated model parameters‡ in the fitting procedure.

Fixed parameters	Baseline value	Range
Average duration of exposed period 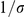	1.9 days (3 stages)	
Average duration of infectious period 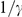		
Sympt. infected	5 days (5 stages)	
Asympt. infected	4 days (4 stages)	
Sympt.* infected	4 days (5 stages)	
Asympt. infected	3 days (4 stages)	
Relative infectiousness		
Sympt. infected (inf_s)	1.0	
Asympt. infected (inf_a)	0.5	
Infectivity profile		
Exposed	0,0,0.25	
Sympt. infected	1.0,1.0,0.5,0.5,0.25	
Asympt. infected	1.0,1.0.0.5,0.5	
Sympt.* infected	1.0,1.0,0.5,0.5,0.25	
Asympt.* infected	1.0,1.0,0.5,0.5	
Antiviral/vaccine efficacy		
infection AE_sus	0.65	
sympt. infection VE_p, AE_p	0.60	
infectiouness VE_i, AE_i	0.15	
**Varied parameters†**		
Proportion sympt. infected, p_s		Beta distr. (a = 3;b = 3)1)
children	0.65	[0.60;0.80]2)
adults,elderly	0.55	[0.5;0.7]2)
Proportion of antivirals used p_use	0.375	0.25;0,0.5
Time delay vaccine effect T_v	7 days	Gamma distr. ( b = 1.5; a = 1;m = 6 d)3)
Vaccine efficacy susceptibility, VE_sus		Beta distr.( a = 3;b = 3)1)
<65 y	0.80	(m = 0.7;n = 0.9)
65+y	0.55	(m = 0.45;b = 65)
Prop. of antivirals prophylaxis p_p	0.04	Uniform; (0.025;0.055)
**Estimated parameters‡**		
Reproductive number (effective) R	fitted	
Relative infectivity of children inf_c	fitted	
Relative susceptibility of children sus_c	fitted	

1)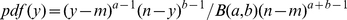.

2) A single beta distribution was used to generate random variates from baseline values.

3)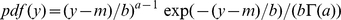.

According to national guidelines, use of antivirals for treatment of clinical influenza should be initiated within 48 hours after symptoms emerge [18]. We modeled this effect by moving symptomatic infected people starting antiviral treatment 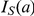 to the less infectious 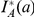 compartments after a mean duration of 24 hours from symptoms emerged (i.e. following the first stage of infection, see Figure 1). The mean duration of the infectious period for this group was assumed to be reduced by 1 day.

### 4. Vaccination

We assumed vaccines to be randomly distributed within each age group. A study among Norwegian health care workers shows that vaccination with adjuvanted A(H1N1) pandemic vaccine elicited a rapid and strong response with 78% and 98% of the vaccinees being protected (HI titres 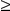
40) after 7 and 14 days, respectively [21]. In the model, vaccinated susceptible individuals were moved to states of vaccine protection 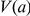 following a time delay of 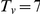 days. Specifically, at time 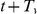 we moved 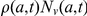 individuals to the vaccinated states, where 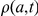 is the density of susceptible individuals in age group 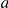 and 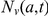 is the number of registered vaccinated individuals at time 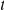. Due to the delay of vaccine effect, we did not consider effects of vaccination among exposed or infected individuals. Results from clinical trials assessing the Pandemrix® vaccine have shown immune response rates close to 100% among adults and children [22], in line with the 98% response rate in the Norwegian study [21]. However, other published studies report a lower vaccine efficacy, and the vaccine efficacy against infection was therefore set to 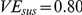 for individuals <65 years, and 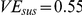 for people 65+ years. The vaccine efficacy for pathogenicity and infectiousness was assumed to be 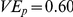 and 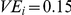. This gives an overall efficacy against symptomatic disease of 0.92 for people <65 years, and 0.8 for people aged 65+ years, which is in line with results from a German study showing a VE of 0.97 and 0.83 in these age groups against laboratory confirmed pandemic infection [22], but higher than results of 0.78 in people <65 years obtained in a pan-European study [23].

We also tested the model with parameters representing a non-adjuvanted influenza vaccine. In these simulations, we assumed vaccine efficacy against infection to be 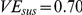 for people <65 years and 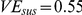 for people 65+ years, and the time delay 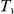 was extended by one week to 14 days [24].

### 5. Estimation of Reproductive Number from Initial Growth Phase Data

The initial growth rate *r* of the weekly ILI data was estimated assuming an exponentially growing epidemic in the weeks 41–44/45. We performed a linear regression of the log-transformed ILI rates and the model fit was evaluated in terms of coefficient of determination. At least 3 weeks of ILI data were used in the estimation procedure, and the choice of numbers of weeks to include was selected based on the goodness of fit.

The data-based reproductive number 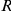 was estimated within a simple *SEIR* framework assuming a latency period of 1.9 days and a mean duration of infectivity of 4.3 days. The reproductive number was calculated from [25]:
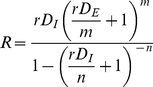
(1.1)where 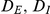 are the mean durations and 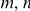 the numbers of stages of the exposed and infectious periods, respectively.

### 6. Model Fitting

We fitted the model by comparing the epidemic curve of the model, i.e. the prevalence of clinical infections, 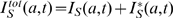, with data of the age-specific numbers of ILI consultations. The model fitting was conducted on daily data from the trough before the major wave (week 41) and the following 13 weeks until the beginning of 2011 (week 53). The comparison was focused on the shape of the epidemic curve or, equivalently, allowing for an undetermined constant of proportionality between number of ILI notifications and cases of influenza. The proportionality was implemented by comparing the transformed quantities 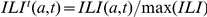 and 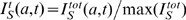. The maxima refer to the overall maximum of symptomatic infected and ILI consultations, respectively, over age groups and time (in practice corresponding to the maximum in the adult population). The model fitting procedure included the following steps:

- A first guess at the real time correspondence of time in the modeled epidemic was made by fixing the time 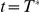 when the density of clinical infections reached 3.5/1000 in the model population as the first day in week 41. This level was chosen based on preliminary simulations.
- Nonlinear least squares was used to obtain estimates for the parameters 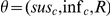 that minimize:
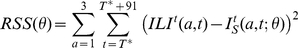
(1.2)
- Step b) was repeated for different choices of the real time anchor within the interval 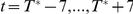. The final model estimate was found by selecting the model in this set with the minimum residual sum of squares.

The optimization was conducted using the *lsqnonlin* function in the Optimization Toolbox in Matlab R2010.

### 7. Scenarios and Sensitivity Analyses

The data on purchased antivirals only provide indirect information on the use of antivirals. In addition, immune individuals may have taken the drugs for prophylaxis, and some people under treatment may have stopped taking the drugs before finishing the recommended treatment period. The parameter 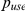 is therefore a composite parameter, incorporating all effects acting to reduce the overall effect of antivirals. Due to uncertainty on the use of antivirals, we considered 3 different scenarios varying 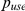 between 25%, 37.5% and 50%.

We performed a probabilistic sensitivity analysis by drawing 500 random sets of parameters representing the time delay for vaccine effect (*T_v_*), the vaccine efficacy on susceptibility (*VE_s_*) and the proportion of antivirals used for prophylaxis (*p_p_*) from the prior distributions described in Table 1. The time delay for vaccine effect was allowed to vary between 6 and 14 days, and the resulting vaccine efficacy against laboratory confirmed infection varied between 0.78–0.96. The model estimation procedurewas conducted for each set of parameters, and for each of the three scenarios 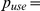
25%, 37.5% and 50%.

## Results

### 1. Epidemiology

**Figure 2:**
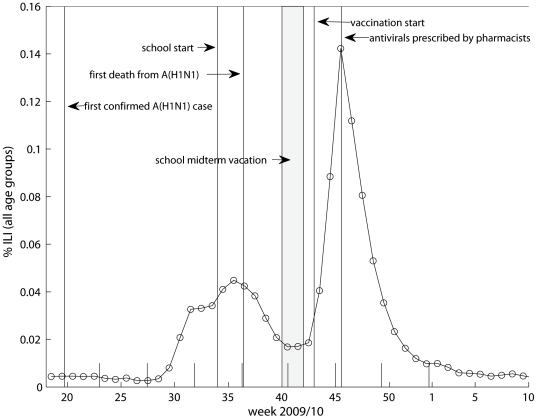
Influenza-like illness (ILI) rates in Norway between May 2009 and February 2010.

**Figure 3:**
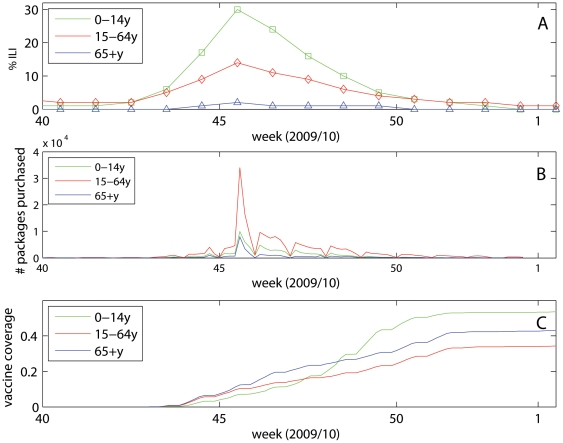
Age-specific Norwegian data on A) influenza-like illness (ILI) , B) purchased antivirals and C) vaccine coverage during the major pandemic wave between October-January 2009/2010.

Figure 2 shows the weekly reported ILI rates between April 2009 and March 2010. The first cases of laboratory confirmed A(H1N1) infection were reported in early May, and continued sporadic spread was observed in the early summer months with few cases in children [13], possibly due to the summer school vacation. In mid-July and August, the ILI activity increased in Norway creating an early summer wave. It is believed that the summer wave was mainly associated with an outbreak of human rhinovirus (HRV), as has also been reported in Sweden and France [26], [27]. A sharp influenza epidemic occurred in October and November with peak in week 45, following shortly after the mid-autumn school vacation (week 40, or week 41). The ILI data suggests high incidence of infection among children and young adults, while elderly seemed to be spared in the pandemic (Figure 3A).

The purchases of antivirals in 2009 amounted to 347,900 packages, of which 39,900 packages were bought for children, 231,280 for adults and 76,720 for elderly people. The vast majority of packages (90% = 311,515/347,900) were sold between week 41 and week 53. On the 5^th^ of November in the midst of the epidemic, Tamiflu® and Relenza® were made available in pharmacies without prescription to ensure easy access and to ease pressure on council health services. This decision led to an immediate increase in the purchase of antivirals, and on that day alone 14.9% (51,883/347,900) of the total 2009 sale of antivirals occurred. While the overall purchase of antivirals seems to follow the epidemic curve (Figure 3B), more antivirals were used in the wake of the epidemic compared to the early phase due to the liberation of prescription policy. No resistance against Oseltamivir and Zanamir was observed by January 2010 [16].

A nationwide vaccination campaign started in week 43 and essentially ended in week 51 of 2009 (Figure 3C), although vaccination continued throughout February 2010. Vaccines were prioritized to health care workers and defined risk groups, followed by a general population-wide vaccination campaign starting with the youngest age groups. The vaccine coverage reached 40% in the population (1.95 million), with 54% coverage in children (0.50 million), and 35% (1.13 million), and 44% (0.31 million) coverage in adults and elderly, respectively.

### 2. Estimation of the Reproductive Number from Data

The fitted growth rates of the ILI curve during the early epidemic with 95% CI were 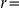
0.069±0.055 per day for the general population, 0.092±0.072 per day for children and 0.059±0.046 per day for adults and elderly, respectively. Based on these numbers, we made a rough estimation of the growth-based reproductive number of the 2009 A(H1N1) epidemic (using Eq. 1), assuming that chemo-pharmaceutical intervention did not interfere with the initial general transmission in the population and that the infectiousness would not vary during the entire infectious period. The calculation gave an estimated reproductive number of 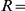
1.35 (1.06–1.69) for the general population with a tendency for higher transmission among children 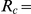
1.48 (1.09–1.97) compared to adults 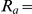
1.29 (1.06–1.57) and elderly 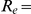
1.29 (1.05–1.56). These numbers should be interpreted with care, however, since the initial spreads in each age group were probably not auto-nomous.

### 3. Model Fitting

**Figure 4:**
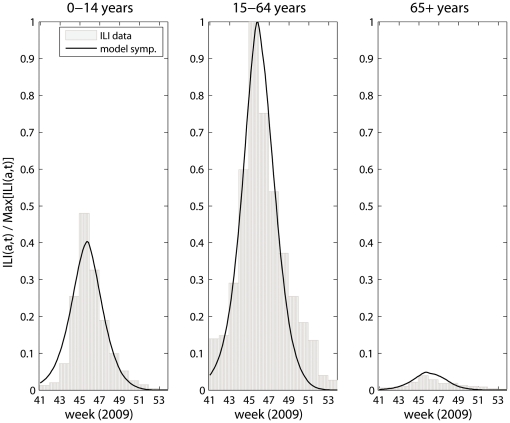
Daily age-specific model fit of symptomatic infections to ILI consultations; right panel: children <15 years, middle panel: adults and left panel: elderly 65+ years, (p_use = 37.5%).

The age-specific fit of symptomatic infections (Figure 4) showed good correspondence with the ILI consultations during the major wave of the pandemic. High transmission levels are seen in children where the ILI consultations reach half the level of adults, which is significantly higher than the population ratio between the two population groups (0.285, based on Norwegian 2010 population data). The fitted relative peak value in children is slightly lower than suggested by data, and the modeled epidemic in the adult population tends to decline faster than the observed ILI curve. This latter finding is not surprising as the model ignores spatial spread and a longer tail would be expected in practice compared to a homogeneous model. Table 2 shows the estimated model parameters (Eq. 2) with residual sum of squares (RSS) for the three baseline scenarios, where the relative use of antivirals compared to the number of purchased packages 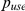 is assumed to be 25%, 37.5% and 50%. The fitted model suggests higher susceptibility and infectiousness in children with 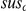 around 1.05–1.06 and infectivity 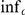 around 1.17–1.20 compared to the remaining population. The reproductive number of the fitted models was estimated at 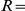
1.37–1.39 in the major pandemic wave. Parameter estimates for models assuming either 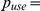
25% or 50% use of purchased antivirals were rather similar, but the best model fit was obtained assuming 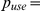
50%.

**Table 2:** Estimated model parameters (relative susceptibility and infectivity in children, and reproductive number), and residual sum of squares (RSS) obtained using nonlinear least squares fitting.

Antiviral use p_use	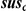	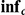	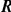	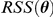
**50.0%**	1.051	1.207	1.371	1.1721
**37.5%**	1.058	1.175	1.388	1.1915
**25.0%**	1.059	1.178	1.392	1.2319

The estimated time from the initial seed of a single infected individual and until the beginning of week 41 varied from 68–71 days, corresponding to transmission starting in late July.

### 4. Impact of Vaccine and Antiviral Based Interventions

**Figure 5:**
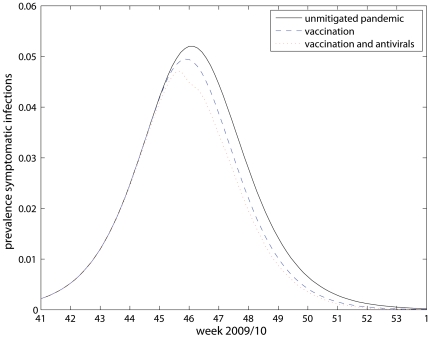
Estimated prevalence of clinical infections during the major autumn/winter 2009 pandemic wave (September–January) for unmitigated pandemic and with pharmaceutical intervention (p_use = 37.5%).

Tables 3, 4 and Figure 5 show the results from the contrafactual simulations of the model using the fitted parameter values in alternative scenarios: 1) with vaccination but without antivirals and 2) assuming an unmitigated pandemic. The model estimates suggest that vaccination and antiviral intervention reduced the overall clinical attack rate in the population from 32–33% to 29–30% (corresponding to an 11–12% reduction of clinical infections). About 75% of the reduction would have been achieved with vaccination alone, thereby accounting for the majority of intervention effects. For the Norwegian population this amounts to 147 000 prevented clinical infections, 110 000 from vaccination alone and 37 000 from antiviral use.

**Table 3:** Estimated age-specific attack rates (symptomatic infections/all infections) during the A(H1N1) pandemic in Norway; antiviral uptake of 25–50% relative to purchased numbers.

		SYMPTOMATIC INFECTIONS					ALL INFECTIONS				
		<15 y	15–64 y	65+y	all	all	<15 y	15–64 y	65+y	all	all
p_use	intervention	%	%	%	%	*1e5	%	%	%	%	*1e5
**50.0%**	no intervention	47,9	34,1	7,7	32,8	16,0	73,7	62,0	14,1	57,2	27,8
	vaccination	45,1	30,8	6,7	30,0	14,6	71,1	56,4	12,3	52,7	25,6
	vaccination+antiv.	43,7	29,5	5,9	28,7	14,0	68,8	53,9	11,2	50,5	24,5
**37.5%**	no intervention	47,9	34,5	7,9	33,2	16,1	73,8	62,8	14,3	57,8	28,1
	vaccination	45,0	31,2	6,8	30,3	14,7	71,1	57,2	12,5	53,3	25,9
	vaccination+antiv.	44,1	30,3	6,3	29,4	14,3	69,6	55,5	11,7	51,8	25,2
**25.0%**	no intervention	48,2	34,7	7,9	33,3	16,2	74,1	63,1	14,4	58,0	28,2
	vaccination	45,2	31,3	6,8	30,4	14,8	71,5	57,4	12,6	53,5	26,0
	vaccination+antiv.	44,6	30,7	6,4	29,8	14,5	70,4	56,2	12,0	52,4	25,5

**Table 4:** Estimated impact of vaccines and antivirals on age-specific total coverage (infection and vaccination) of the A(H1N1) influenza, and estimated intervention impact on timing and maximum prevalence of symptomatic infections.

		COVERAGE					PEAK		
		INFECTIONS/VACCINATIONS					SYMPTOMATIC INFECTIONS		
use of antivirals	intervention	<15 y	15–64 y	65+y	all	all	ΔT_peak	Max I_s	Max I_s
p_use		%	%	%	%	*1e5	days	*1e5	%
**50.0%**	no intervention	73,7	62,0	14,1	57,2	27,8	0,0	2,5	100,0
	vaccination	84,5	70,7	22,3	66,2	32,2	−1,0	2,4	96,6
	vaccination+antiv.	83,1	68,9	21,5	64,6	31,4	−3,0	2,2	89,9
**37.5%**	no intervention	73,8	62,8	14,3	57,8	28,1	0,0	2,5	100,0
	vaccination	84,7	71,3	22,5	66,7	32,4	−1,0	2,4	96,6
	vaccination+antiv.	83,7	70,2	22,0	65,7	31,9	−3,0	2,3	91,5
**25.0%**	no intervention	74,1	63,1	14,4	58,0	28,2	0,0	2,5	100,0
	vaccination	84,9	71,8	22,8	67,0	32,6	−1,0	2,4	96,5
	vaccination+antiv.	84,1	70,7	22,2	66,1	32,1	−3,0	2,3	92,4

The estimated numbers of subclinical infections are in the range 1.05–1.09 million cases, and the number of people who had experienced infection (whether symptomatic or asymptomatic), or who had been vaccinated amount to 64–65% of the entire population by the end January 2010 (Table 4). In this calculation, elderly people with assumed prior immunity, which comprise approximately 8% of the total population, are not included. The simulations suggest that the natural peak of the pandemic would have been close to the observed peak, as the peak occurred approximately 3 days earlier due to pharmaceutical interventions. However, the combined effect of vaccines and antivirals lowered the estimated peak prevalence of symptomatic infections by 9–10% compared to the unmitigated pandemic. Vaccination alone would have reduced the peak by 3–4%, only, suggesting that the release of antivirals in week 45 may have had a significant impact on the epidemic peak.

### 5. Sensitivity Analyses

The general impression from the sensitivity analysis is that the conclusions from the main scenario are quite stable. For simplicity, all intervals reported below refer to the central 95% of the distributions of values that were generated using the 500 random parameter sets as described in Methods, section 6.

The model reproductive number varied within the range of [1.32, 1.39] assuming 50% effective AV use, within [1.36, 1.40] for 37.5% AV use and within [1.37, 1.42] for 25% AV use. The respective means were 1.38, 1.39 and 1.39. The two other fitted parameters; relative susceptibility and infectivity in children, varied even less.

For the relative susceptibility, the three means were 1.05, 1.06 and 1.06, respectively, with 95% intervals covering less than ±0.03 around the means. For the relative infectivity, the three means were 1.19, 1.18 and 1.18, respectively, with slightly larger 95% intervals, all less than ±0.06 around the mean.

In all 500 parameter sets, the 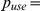
50% assumption lead to a better fit than the 37.5% and 25% alternatives. The effects on the simulated scenarios (no intervention, only vaccination, vaccination and antivirals) and on the related measures of efficacy are also relatively stable. As an example in the 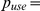
50% scenario, the clinical attack rate varied within the range of [0.28, 0.33] and the reduction achieved by vaccination+antivirals within [0.03, 0.05], with means 0.31 and 0.04, respectively. The total attack rate (including asymptomatic infections) varied within [0.55, 0.58] and the reduction in [0.03, 0.07], with means 0.57 and 0.06, respectively. Thus, within the limits of the performed parameter variations, our main scenario appears to be rather central, with moderate possible deviations.

### 6. Timing of Vaccination Start

**Figure 6:**
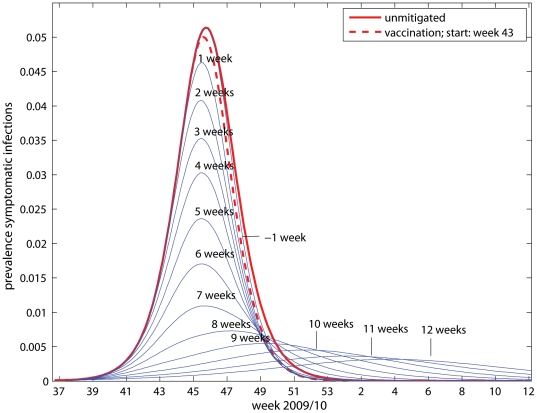
The effect of timing of vaccination start on the pandemic curve. The unmitigated pandemic is shown with thick line; no use of antivirals was implemented in these simulations (p_use = 0%).

**Figure 7:**
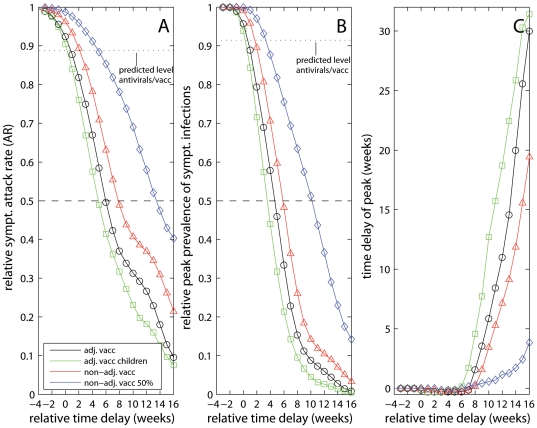
The effect of timing of vaccination start on A) pandemic clinical attack rate, B) peak prevalence of symptomatic infections and C) time delay of peak relative to unmitigated pandemic; no use of antivirals implemented in these simulations (p_use = 0%). For comparison, the predicted relative reduction in the baseline scenario with vaccine and antivirals is shown (delay = 0 week) in A and B.

To study the effect of vaccination timing on influenza transmission, we performed simulations where the vaccination campaign (that started in week 43) was assumed to commence at various times between week 27 and week 45. In these simulations no use of antivirals was incorporated, and the vaccination schedule was assumed to match the Norwegian data except for the shift in time. Figure 6 shows the estimated prevalence of symptomatic influenza cases during the course of pandemic that would have been observed had vaccination been initiated at different points in time. Figure 7A shows the relative clinical attack rate compared to that of an unmitigated pandemic as function of the vaccine start. It is seen that the additional preventive effect of antivirals could have been accomplished by adjuvanted vaccines alone, had the campaign started half a week earlier (black line). Vaccination should have commenced approximately 6 weeks earlier to achieve a 50% reduction in the clinical attack rate, and 12.5 weeks earlier to reduce the attack rate by 75%. Figure 7B depicts the relative peak prevalence of symptomatic infections compared to the maximum level of an unmitigated pandemic as function of the timing of the vaccine start. Vaccination came too late to have any significant impact on the peak number of infections. If vaccination had begun one week later, the pandemic would have passed its natural peak before the vaccines would have started to show effect. It is seen that a 50% reduction in the peak level would have been achieved, had vaccination commenced 4.5 weeks earlier than the actual start. Figure 7C shows the estimated time delay of the pandemic peak relative to the peak of the unmitigated pandemic as function of the timing of the vaccine start. It is seen that vaccination should have commenced at least 8 weeks earlier in order to delay the peak of the pandemic by half a week, and 10.5 weeks earlier to achieve a peak delay of 8 weeks.

### 7. Adjuvanted Vaccines Prioritized for Children

We simulated a vaccination campaign using adjuvanted pandemic vaccines prioritized for children, followed by adults and elderly (Fig. 7, green lines). The final coverage in the age groups and daily vaccinations were assumed identical to data. In this instance, vaccination of children ended after 22 days, and vaccination of adults was completed 54 days into the program. A prioritized vaccination to children starting in week 43 would have prevented an estimated extra 47,200 symptomatic infections (1.7%) compared to the adopted vaccination scheme in Norway. Vaccination should have been initiated 5 and 9.5 weeks earlier to achieve a 50% or 75% reduction in the attack rate, equivalent to a gain of one week and 3 weeks, respectively, compared to the corresponding values calculated using the actual vaccination strategy (Fig. 7A). Correspondingly, a 50% reduction in the peak level was predicted to occur, had vaccination started 3.5 weeks ahead of time, representing a gain of 1 week compared to the actual vaccination scheme.

### 8. Non-Adjuvanted Vaccines

Finally, we used the model to explore the mitigating potential of a non-adjuvanted vaccine adopting a vaccination scheme equivalent to the actual one (Fig. 7, red lines), and by assuming a 50% reduction of the vaccination rate (Fig. 7, blue lines) as use of non-adjuvanted vaccines may not only affect the timing of vaccination start, but also potentially reduce the rate of vaccine delivery thereafter. Vaccination with non-adjuvanted vaccines starting in week 43 would have resulted in an estimated 108,600 additional symptomatic cases (3.9%), while 172,800 extra symptomatic cases (6.1%) were predicted if the rate of vaccine delivery is reduced by 50% compared to data. Even a slight delay in the vaccination start would have reduced the effect of vaccination to practically nothing. A reduction in the attack rate of 50% or 75% would have been achieved with a non-adjuvanted vaccine, if the intervention had started 8 or 15 weeks earlier, exceeding the estimates for the adjuvanted vaccine by 2–2.5 weeks (Fig. 7A). In the case of a 50% reduction in vaccine delivery, vaccination should have commenced 13.5 weeks earlier to achieve a 50% reduction in the attack rate, equivalent to further 7.5 weeks compared to the earlier start needed with full delivery of adjuvanted vaccine.

## Discussion

The present study highlights the critical issue of vaccination timing in relation to pandemic spread. In Norway, vaccination started too late to have a strong impact on the 2009 A(H1N1) pandemic. Our results suggest that adjuvanted vaccines in combination with antivirals lowered the clinical attack rate by an estimated 11–12% in relative terms (3–4% in absolute terms). Vaccination was found responsible for approximately 3 in 4 prevented infections. Our study indicates that had vaccination started 6 weeks earlier, the clinical attack rate would have been reduced by 50%, while the vaccine effect would have been reduced to almost nothing, had the intervention started only 2 weeks later.

The vaccination strategy adopted by the Norwegian public health authorities during the 2009 pandemic was aimed at protecting risk groups, rather than to affect the overall course of the epidemic. Our results show elevated transmission among children, in particular driven by higher infectiousness compared to adults and elderly. We found that a vaccination campaign prioritized for children with similar coverage and rate of vaccination as in the Norwegian data would have been effective approximately one week earlier compared to the strategy adopted, when measuring the ability to reduce the overall attack rate by 50%. However, as vaccination began in week 43 in the midst of the pandemic, the additional benefit from vaccinating children first would have been limited (1.7% relative reduction). Clearly, there are ethical aspects to consider in relation to prioritizing vaccines for children, as reduced transmission comes at the cost of higher risk of severe disease and death among frail individuals in the population. Therefore, inclusion of children in the priority group for vaccination must be weighed against the severity of the influenza, and may be indicated, for instance in the case of a potential future pandemic with an avian (H5N1) virus.

Widespread use of adjuvanted influenza vaccines is rather recent. These vaccines are advantageous compared to conventional vaccines because each vaccine dose contains a smaller amount of antigen, thereby reducing the vaccine production time. Compared to the non-adjuvanted vaccines most commonly used routinely for seasonal influenza, we found that adjuvanted vaccines were effective approximately 2 weeks earlier, measured by their capacity to reduce the attack rate by 50%. These results ignore any potential time delay in the delivery of non-adjuvanted vaccines, and hence, the mitigating potential of non-adjuvanted vaccines during the 2009 pandemic in Norway would likely have been very limited. Norway was among the countries in Europe with the most liberal policy of administration of antivirals during the 2009 (H1N1) pandemic. Although our estimates indicate that antivirals had a minor impact on the spread of influenza, we find it plausible that the policy change in week 45 allowing pharmacists the right to distribute antivirals may have contributed to slowing the transmission in the weeks around the pandemic peak.

Our model suggest a reproductive number in the early phase of the major pandemic wave of approximately 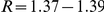, which is close to the estimate of 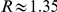 that we obtained using the initial growth rate of the ILI data. Both estimates are consistent with a median reproduction number of 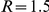 reported in a review study [28], and with a previous UK estimate of approximately 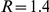 [29]. Our results indicate that around 72–73% of the population were either infected or vaccinated during the course of the major pandemic or possessed an assumed pre-pandemic immunity against the A(H1N1) virus. Norwegian serological data from January 2010 showed that 60% of the population had reactive antibodies against the pandemic virus [13]. However, the interpretation of the serological data is complicated and antibody levels are only partly a marker for immunity, e.g. not taking into account cellular immunity. For instance, the serological data showed that 28% (95% CI 18–38%) of people aged 50–64 years had antibodies to the 2009 A (H1N1) virus, despite a vaccine coverage of 43% in this age group. Thus the results from our study may be considered as compatible with the serological results. Our estimate of 1.40–1.45 million clinical cases is in the higher end of the current official Norwegian estimate of 0.45–1.8 million symptomatic infections [30], which is based on assumptions that 5–20% of people with influenza-like illness contacted the health care system. However, there is uncertainty surrounding these estimates, and there is a fluent transition between mild symptomatic and asymptomatic infection, particularly since the A(H1N1) is considered to have been a mild pandemic. A recent UK based study assumed that only 35% of A(H1N1) infections were symptomatic [31]. When re-fitting our model assuming 45% symptomatic cases among children and 35% symptomatic cases among adults/elderly, we obtained parameter estimates of 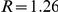, 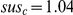 and 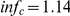 (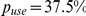), with a corresponding clinical attack rate of 1.0 million clinical cases (21%), while 76% of the population were either infected or vaccinated during the course of the pandemic, or were naturally immune. The latter estimate is slightly higher than our baseline estimate of 72% (full data sheet is available from the authors upon request). One limitation of our study is that we assumed that the proportion of people with influenza seeking medical care was age- and time-independent during the major wave, and this may have biased our estimates of the relative susceptibility and infectivity in children. It is possible that the attitude towards seeking GP assistance during the 2009/2010 season was different due to the pandemic scare compared to normal influenza season. In addition, the Norwegian government and social partners implemented a common policy on October 23 (week 43) by extending the self-certification period of sickness absence due to A(H1N1) influenza from 3 to 8 days. However, no data was available on the attitude towards seeking GP treatment for influenza, and therefore it was not considered in the model. Another restriction is the lack of spatial structure in the model, which may explain the slightly steeper decline in the fitted model compared to the sentinel ILI data. However, the timing of the pandemic peak in different regions of Norway varied little and was of the order of 1 week.

In conclusion our results underscore the critical role of timing of the vaccination campaign and the importance of fast and efficient delivery of vaccines. Our results also corroborate previous findings showing that children played a pivotal role for the spread of the pandemic.
